# Long-Term Safety Following Faecal Microbiota Transplantation as a Treatment for Recurrent *Clostridioides difficile* Infection Compared with Patients Treated with a Fixed Bacterial Mixture: Results from a Retrospective Cohort Study

**DOI:** 10.3390/cells11030435

**Published:** 2022-01-27

**Authors:** Frederik Cold, Camilla Kara Svensson, Andreas Munk Petersen, Lars Hestbjerg Hansen, Morten Helms

**Affiliations:** 1 Gastro Unit, Medical Division, Copenhagen University Hospital Hvidovre, Kettegaards Alle 30, 2650 Hvidovre, Denmark; frederik.cold@regionh.dk (F.C.); andreas.munk.petersen@regionh.dk (A.M.P.); 2 Department of Plant and Environmental Sciences, Faculty of Sciences, Copenhagen University, Thorvaldsensvej 40, 1871 Frederiksberg, Denmark; lhha@plen.ku.dk; 3 Department of Infectious Diseases, Copenhagen University Hospital Hvidovre, Kettegaards Alle 30, 2650 Hvidovre, Denmark; camilla.kara.svensson@regionh.dk; 4 Department of Clinical Microbiology, Copenhagen University Hospital Hvidovre, Kettegaards Alle 30, 2650 Hvidovre, Denmark; 5 Department of Clinical Medicine, Faculty of Health and Medical Sciences, Copenhagen University, Blegdamsvej 3B, 2200 Copenhagen, Denmark

**Keywords:** FMT, rectal bacteriotherapy, safety, long-term, faecal microbiota, survival, IBD, gut microbiome, multidrug-resistant organism

## Abstract

Faecal microbiota transplantation (FMT) is the recommended treatment for recurrent *C. difficile* infection (rCDI) following a second recurrence. FMT is considered safe in the short term when procedures for the screening of donors and transferred material are followed. However, the long-term safety profile of FMT treatment is largely unknown. In a retrospective cohort study, we assessed the long-term safety of patients treated for rCDI with FMT or a fixed bacterial mixture, rectal bacteriotherapy (RBT). The overall survival, risk of hospital admission, onset of certain pre-specified diseases (cancer, diabetes mellitus, hypertension and inflammatory bowel disease) and risk of being diagnosed with a multidrug-resistant organism were assessed by undertaking a review of the treated patients’ medical records for up to five years following treatment. A total of 280 patients were treated for rCDI with FMT (*n* = 145) or RBT (*n* = 135) between 2016 and 2020. In the five years following treatment, there were no differences in survival (adjusted hazard ratio (aHR) 1.03; 95% CI 0.68–1.56), *p* = 0.89), risk of hospital admission ((aHR 0.92; 95% CI 0.72–1.18), *p* = 0.5) or onset of any of the analysed diseases. In conclusion, FMT was not associated with increased mortality, risk of hospital admission or onset of disease following treatment when compared with RBT.

## 1. Introduction

*Clostridioides* (formerly *Clostridium*) *difficile* is a spore-forming bacteria that, under certain conditions, releases toxins, resulting in infectious diarrhoea. The primary treatment of *C. difficile* infection (CDI) is antibiotics. In the case of recurrent *C. difficile* infection (rCDI), treatment with faecal microbiota transplantation (FMT) from healthy faecal donors has been shown to be superior to antibiotics and is currently the recommended treatment option following a second recurrence of CDI [1,2,3]. FMT as a treatment of rCDI has been reported to have high cure rates, regardless of whether the treatment is delivered through upper or lower endoscopy, enemas or capsules [4]. FMT is also being investigated as a treatment for other diseases where an altered gut microbiome has been found to be correlated with disease, such as altered glucose metabolism, hypertension, inflammatory bowel disease (IBD) and certain types of cancer [5,6,7,8,9]. Furthermore, FMT is being investigated as a treatment of multidrug-resistant organisms (MDRO) [10]. Primarily in the treatment of IBD and MDRO, the results of treatment with FMT are promising, although further randomised controlled trials are needed [10,11,12,13].

When guidelines for the screening of donors and faecal material are followed, FMT treatment is regarded as safe in the short term, with only transient, mainly gastrointestinal side effects and risks from endoscopic procedures [14,15]. Thus, FMT is generally considered safe [16]. However, rare cases of transfer of MDRO through FMT have been reported [17,18]. Furthermore, cases of potential worsening or onset of IBD have been reported following FMT, although this has not been reproduced in randomised controlled trials (RCTs) [19,20,21]. Based on animal studies, there are also concerns that FMT treatment manipulating the gut microbiome could potentially induce other conditions such as cancer and metabolic diseases [22]. Information concerning long-term risks following FMT is currently limited [15,23,24,25]. Patients receiving FMT for rCDI are often multimorbid with reduced life expectancy, and it is therefore difficult to measure the long-term risks following FMT [25]. Since FMT is superior to a placebo and antibiotics in the treatment of rCDI and reduces mortality, it is unlikely that there will be future RCTs comparing FMT with a placebo or antibiotics [4,26,27].

At Copenhagen University Hospital Hvidovre (CUHH), in addition to FMT, rectal bacteriotherapy (RBT) is also used in the treatment of rCDI. RBT is a defined mixture of 12 bacterial strains, with reported cure rates of 52–88% in the treatment of rCDI [26,28,29]. No studies have reported long-term changes in the gut microbiome or in long-term safety following RBT [28]. The risk of transferring harmful microbes or traits is potentially higher when transferring a full faecal microbiome through FMT, including potential unknown harmful microorganisms (bacteria, fungi, protozoa, archaea and virus), than when transferring a fixed bacterial cocktail through RBT.

The working hypothesis of this study was that the risk of transfer of harmful traits is higher when FMT is used in the treatment of rCDI compared with RBT. By undertaking a long-term follow-up for up to five years of patients treated with FMT or RBT, the aim of this study was to assess whether patients receiving FMT had a greater risk of dying, being admitted to hospital or being diagnosed with a new onset of certain predefined diseases than patients treated with RBT. Furthermore, a secondary aim was to assess whether FMT treatment induced beneficial effects, with a greater chance of no longer needing to treat the same pre-specified diseases.

## 2. Materials and Methods

### 2.1. Treated Patients

All patients in the Capital Region of Denmark presenting with recurrent or refractory CDI and for whom FMT or RBT are considered treatment options are referred to the Department of Infectious Diseases at CUHH. In this retrospective cohort study, patients of all ages, including children, treated with FMT or RBT outside randomised clinical trials from 1 January 2016 to 31 December 2020 were included. The follow-up concluded on 5 May 2021. Safety data from 98 patients included in a randomised clinical trial (May 2017–March 2019), in which CUHH was one of the trial sites, comparing FMT delivered through enema, RBT and vancomycin are reported elsewhere and are not included in this dataset [26].

At CUHH, FMT delivered through various routes of administration (capsules, enemas or upper or lower endoscopy), RBT and antibiotics are used in the treatment of recurrent, refractory or severe CDI [26]. The decision as to whether patients are treated with FMT delivered through endoscopy, enemas, capsules, RBT or antibiotics for their current episode of CDI is taken based on patient preference, the physician’s clinical decision and the current status of available treatments.

Recurrent CDI is defined as diarrhoea with the passage of ≥3 loose or liquid stools per day and a faecal sample positive for *C. difficile* (a positive polymerase chain reaction test result for CD toxin A, toxin B or binary toxin) after a former episode of CDI treated with Vancomycin, Metronidazole or Fidaxomicin, with clinical resolution (absence of diarrhoea or negative stool sample for CD) achieved during treatment. Exclusion criteria for FMT treatment of CDI at CUHH are concurrent gastrointestinal infection with another pathogenic microorganism that must be treated prior to potential FMT/RBT, or current antibiotic treatment for other diseases, except prophylactic antibiotic treatment. All the treated patients were monitored for at least eight weeks in telephone interviews conducted by a medical doctor.

### 2.2. Treatments

Patients were treated according to guidelines at the time of treatment, including pre-treatment with the oral antibiotics Vancomycin (4–10 days of 125/500 mg × 4 daily) or Fidaxomicin (4–10 days of 200 mg × 2 daily) prior to FMT or RBT [2,30]. Antibiotic treatment was stopped 12 h before RBT and 24–36 h prior to FMT, irrespective of the route of administration.

#### 2.2.1. Rectal Bacteriotherapy

The bacterial mixture for rectal bacteriotherapy consisted of 12 well-characterised gut bacterial strains, previously described elsewhere [28,31]. The bacteria were originally isolated from the faeces of healthy volunteers. The bacterial mixture was produced on each day of the treatment and transported directly to the study site. RBT is considered a drug, produced in accordance with Good Manufacturing Practice principles and approved and monitored by the Danish Medicines Agency (Drug id: 27415700915) [26]. It is delivered as an enema by a catheter (Ch 12, diameter 4 mm) inserted 30–40 cm rectally with the patient in the left lateral position with bent hips and knees during the procedure and for one hour afterwards. In individual cases, RBT can be delivered through a stoma. The treatment is delivered for three consecutive days.

#### 2.2.2. Faecal Microbiota Transplantation

FMT provided by various Danish FMT stool banks was used for different periods at CUHH, based on availability. Up to September 2017, only fresh donor material was used. Following a period using both fresh and frozen donor material, with effect from January 2018, all FMT treatments were from frozen donor material.

Throughout the study period, FMT delivered through enemas or endoscopy produced from the FMT stool bank at CUHH was used. FMT delivered through enemas was administered in the same way as RBT, and as a single treatment on a single day. In 2016 and 2017, FMT donors were either relatives (*n* = 22) or unrelated universal donors recruited from the Danish blood donor corps (*n* = 29). From 2018, only unrelated universal donors were used. Both groups were tested in accordance with international guidelines, as described in previous publications [29,30,32]. FMT material for stomal or nasojejunal delivery was screened and produced at the FMT laboratory at CUHH following the same procedures. The amount of stool used in the production of all (enema, stoma or nasojejunal) treatments varied from 50 to 200 g.

From July 2019 onwards, FMT delivered through enemas or endoscopy from the Centre for Faecal Transplantation (CEFTA) at Aarhus University Hospital was also used. FMT from CEFTA was administered on a single day through an enema or endoscopy, in accordance with the previously described procedure. A single treatment was derived from approximately 50 g of crude faecal material [33].

The FMT capsules administered during this period came from two different Danish FMT stool banks. Capsules from the Aleris-Hamlet FMT stool bank were produced in two different ways: from October to December 2018, they were multi-donor FMT capsules, as described by Chehri et al. [34], and from October to December 2020, they were single-donor FMT capsules, all from the same donor. Both single- and multi-donor capsules were distributed on three consecutive days, 25 capsules per day. A three-day FMT capsule treatment was produced from a total of 150 g of crude faecal material. From September 2019 onwards, FMT capsules from the CEFTA at Aarhus University Hospital were used [35]. The capsules were administered only once and consisted of 15–30 single-donor FMT capsules produced from approximately 50 g of crude faecal material.

### 2.3. Recorded Data

All the data used in this study were based on a review of the patients’ electronic medical records, with data recorded in real time at all public hospitals and hospital departments in the Capital Region of Denmark and Zealand Region. The data were evaluated and recorded from January to May 2021. Patients’ contacts with hospitals in the two regions from January 2016 to May 2021 were reviewed.

The following data were recorded at the time of first treatment with either FMT or RBT for the current episode of rCDI: date of treatment, age at treatment, previous treatment of CDI, number of recurrences of CDI and Charlson Comorbidity Index. Furthermore, the presence of MDRO or any of the following diseases was recorded at the time of FMT or RBT treatment: hypertension, inflammatory bowel disease (IBD), diabetes mellitus and cancer. Patients who died during the follow-up period were censored from the time of death for all other outcomes. Patients who were alive during the follow-up period were censored from the day of registration. At each new hospitalisation, the patient’s medication list was reviewed for the purposes of evaluating the potential disappearance of disease/cessation of treatment.

In the review of patients’ electronic medical records, it was not possible to determine whether patients had moved to a different Danish region or to another country, leading to a risk of not being able to follow up with patients if they had relocated.

Data on clinical resolution following FMT or RBT were outside the scope of this study. All included data were reviewed by two investigators (F.C. and C.K.S.) and, in the event of disagreements, M.H. was consulted.

#### 2.3.1. Survival

The date of death was noted by reviewing patients’ medical records. Time of death is registered in the patient’s medical records with a 24–48 h delay by cross-referencing with the Danish civil registration system.

#### 2.3.2. Onset of Disease

Each time a patient was hospitalised, the onset or disappearance of disease was evaluated. The onset of disease was registered as the first day on which this was registered in the patient’s medical record, either through registration of the disease by a physician or through administration of medication to treat the disease (see sections below). Onset of disease was only registered in patients not registered with the disease at the time of FMT/RBT treatment. All data were collected retrospectively; hence, treating physicians were not systematically looking for the onset of the pre-specified diseases or testing for the presence of MDRO following treatment. The decision to include the selected diseases was based on previously published data concerning potential beneficial or harmful effects of FMT and the availability of data to evaluate the onset or disappearance of disease through the patients’ medical records.

#### 2.3.3. Disappearance of Disease/Cessation of Treatment

The disappearance of a disease or cessation of treatment for a disease were recorded as the first day on which this was entered in the medical records of patients for whom the disease was registered at the time of FMT or RBT. Disappearance of disease or cessation of treatment for disease were recorded if this was described in the patient’s medical record by a treating physician or through registration of no medication to treat the disease (see sections below).

#### 2.3.4. Cancer

Patients were recorded as having cancer at the time of FMT or RBT if they were diagnosed with an active cancer, other than non-melanoma skin cancer, that was newly diagnosed; if they were receiving anti-cancer treatment; or if they were receiving no treatment due to the absence of further treatment for their cancer. Patients who were part of a follow-up scheme for a previous cancer, without ongoing disease or treatment, were not recorded as having cancer at the time of FMT or RBT. A new cancer diagnosis was defined as the presence of a new primary cancer. A recurrence of a previously diagnosed cancer was not recorded. The day on which a clearly defined diagnosis of cancer was entered was defined as the day of onset of a new cancer. Disappearance of cancer was recorded in patients who were registered with cancer at time of FMT or RBT who stopped cancer treatment without ongoing treatment and for whom there was a description of no active cancer disease in the patient’s medical records. All cancer treatment in Denmark is given at public hospitals.

#### 2.3.5. Diabetes Mellitus, Hypertension and Inflammatory Bowel Disease

The presence of diabetes mellitus was identified by active treatment of the disease based on a diagnosis in the patient’s medical record or treatment with the following antidiabetic medications: biguanides, sulfonylureas, DPP-4 inhibitors, GLP-1 receptor agonist, thiazolidinediones or insulin. In the case of treatment with insulin, the type of diabetes registered was based on the physicians’ entry in the patient’s medical record. All Danish patients diagnosed with type 1 diabetes mellitus are initially treated in a hospital department. Patients with type 2 diabetes mellitus in Denmark are treated both in hospital departments and by general practitioners. The onset of diabetes mellitus was registered as the first day on which this was registered in the patient’s medical record, either through registration of the disease by a physician or through administration of one of the mentioned drugs.

The presence, onset and disappearance of hypertension and IBD were based on the same principles as for diabetes mellitus (Supplementary Material, methods section).

#### 2.3.6. Multidrug-Resistant Organisms

The presence of MDRO prior to FMT or RBT treatment was based on its presence in a blood sample, faecal swab or sample, or urine sample that was registered as being multidrug resistant. Patients were registered as having an MDRO if they had had a positive test within the previous three months with no subsequent negative test prior to FMT or RBT. Onset of MDRO following treatment was based on the presence of an MDRO in the first blood sample, faecal swab or sample, or urine sample within six months of FMT or RBT, not preceded by samples without this MDRO. Disappearance of MDRO was based on its absence or loss of multidrug resistance through a new blood sample, faecal swab or sample, or urine sample following FMT or RBT treatment in patients registered with an MDRO at the time of the treatment. All bacterial samples analysed in Danish hospitals are analysed for drug resistance, although exact procedures may vary from hospital to hospital.

#### 2.3.7. Day of First Hospital Admission Following Treatment

The day of the first hospital admission following FMT or RBT treatment was recorded. Scheduled hospital admissions due to procedures that were planned prior to FMT or RBT treatment or ongoing follow-up of diseases diagnosed prior to FMT or RBT were not registered.

### 2.4. Statistical Analyses

Patients receiving FMT or RBT were analysed and divided into two groups based on the first treatment they received for their episode of CDI. All patients receiving FMT were registered as a single group, regardless of the route of administration. Patients who received both FMT and RBT for their episode of CDI were registered according to the first treatment. Patients admitted to hospital (*n* = 3) who were receiving treatment for fulminant CDI (fever ≥ 38.5°C, mental disorientation, hypotension, ileus, megacolon, pseudomembranous colitis, multi-organ failure) were excluded from the analyses. Patients presenting with a new recurrence of CDI following clinical resolution (absence of diarrhoea and/or stool test negative for CD for eight weeks) following FMT or RBT treatment who were being re-treated were not registered again.

Cox proportional hazards regression models were performed comparing survival, onset of disease and risk of hospitalisation in the FMT group versus the RBT group. The effects of potential confounders (number of CDI recurrences, age, gender and Charlson Comorbidity Index score) were evaluated in the models by adding a variable to the unadjusted model and comparing the hazard ratio (HR) and confidence intervals (CI) of this model with the HR and CI from the unadjusted model. If the change was sufficiently large, the variable was considered a confounder. This was repeated for all possible confounders. Subsequently, an adjusted model including all confounders was fitted. For the survival analysis and risk of hospitalisation analyses, HRs were adjusted for age, Charlson Comorbidity Index score and gender. For the FMT versus RBT comparison of the risk of being diagnosed with the onset of a new disease, adjusted models were not run due to the small number of events (*n* ≤ 20). The following subgroup analyses were undertaken in the comparison of survival between the groups: (i) subgroup analyses excluding patients receiving previous treatment with FMT or RBT; (ii) subgroup analysis excluding patients receiving treatment with both FMT and RBT for their current episode of CDI; (iii) subgroup analysis omitting patients receiving treatment with both FMT and RBT either for a current or previous episode of CDI; (iv) subgroup analysis omitting patients who at any time received treatment with both FMT and RBT for a previous, subsequent or current episode of CDI; (v) subgroup analysis comparing only patients receiving FMT delivered through an enema with RBT delivered through an enema; (vi) subgroup analysis comparing patients receiving FMT delivered through an enema with patients receiving FMT delivered through capsules.

Gray’s test was used to test the difference in the chance of disappearance of a disease/cessation of treatment between the FMT- and RBT-treated patients throughout the follow-up period. The difference in the proportion of patients diagnosed with a certain disease at baseline or at a certain time during the follow-up after FMT or RBT was assessed through the Chi-square test for large sample sizes or Fisher’s exact test for small sample sizes with fewer than five expected counts. The difference in mean values of continuous variables at baseline was analysed through t-tests. All calculations were performed using R version 4.0.3 including the “survival”, “prodlim” and “timereg” packages.

## 3. Results

**Figure 1:**
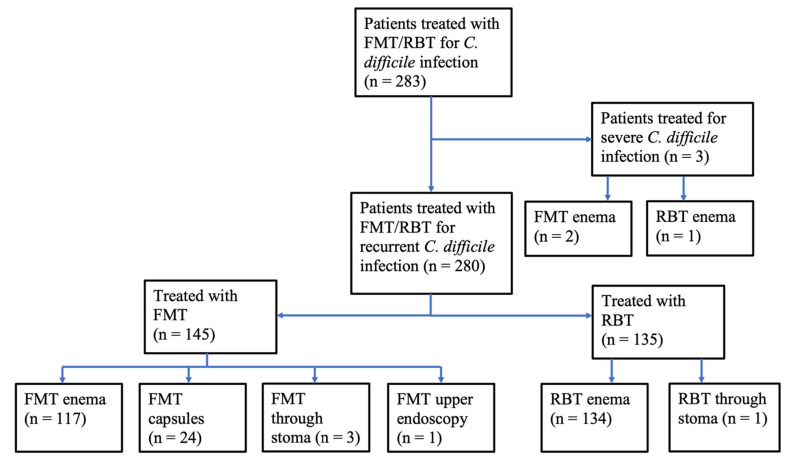
Patients treated with FMT or RBT for *C. difficile* infection. Primary treatment of recurrent or refractory *C. difficile* infection at Copenhagen University Hospital Hvidovre 2016–2020. Faecal microbiota transplantation, FMT; rectal bacteriotherapy, RBT.

Between 2016 and 2020, a total of 283 patients were treated at CUHH for recurrent or refractory CDI with FMT or RBT as the primary treatment following antibiotics (Figure 1). Among the 145 patients receiving FMT delivered through an enema, stoma or endoscopy, 24 received FMT from family donors (child (*n* = 14), grandchild (*n* = 3), sibling (*n* = 4), other relative (*n* = 3)), while 121 patients received FMT from anonymous donors. All FMT capsules were from anonymous donors.

There was no difference in age, sex, number of CDI recurrences and previous treatment with FMT or RBT between the FMT group and the RBT group (Table 1). Furthermore, there was no difference in co-morbidities assessed by the Charlson Comorbidity Index. Type-1 diabetes mellitus, which was significantly more prevalent in the RBT group, was the only disease that was more prevalent in one group than the other. FMT was the only treatment that was delivered to patients in their home. More patients were hospitalised in order to receive RBT than FMT because the RBT treatment was given for three consecutive days. The reason why patients were hospitalised for the treatment was mostly due to the long distance from their home to the hospital. More patients primarily treated with RBT also received a mixed treatment with FMT for the same episode of CDI following unsuccessful treatment.

### 3.1. Survival

**Figure 2:**
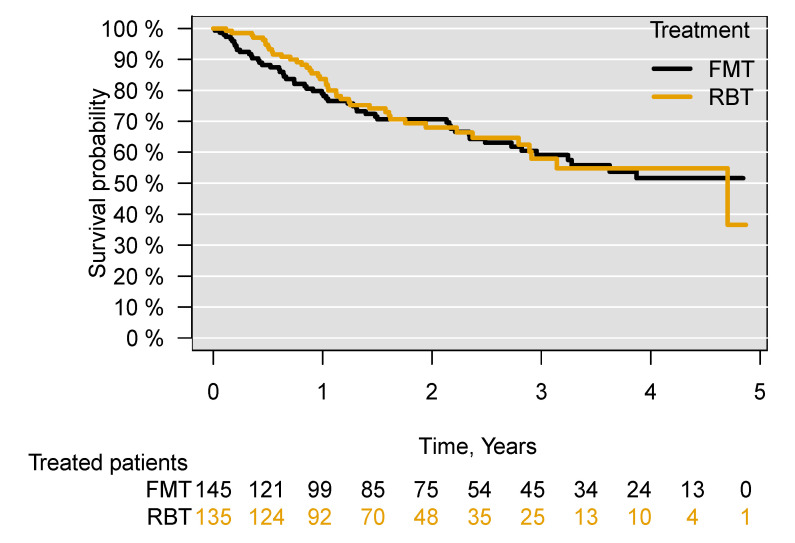
Survival following treatment of recurrent *C. difficile* infection with FMT or RBT through all routes of administration. Faecal microbiota transplantation, FMT; rectal bacteriotherapy, RBT.

The median follow-up period of treated patients was 603 days. Among the 95 patients who died in the five years following treatment, the median time to death was 356 days. There was no difference in survival between patients treated with FMT and those treated with RBT ((aHR = 1.03; 95% CI 0.68–1.56), *p* = 0.89) in the five years following treatment (Figure 2).

**Figure 3:**
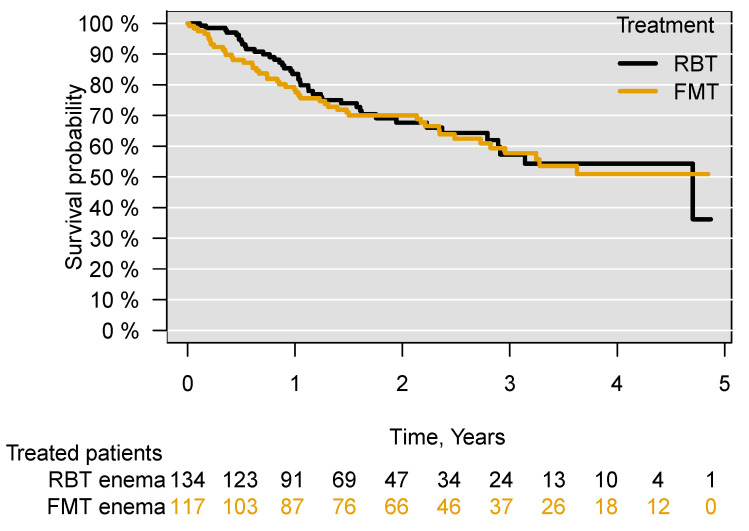
Survival following treatment of recurrent *C. difficile* infection with FMT or RBT through an enema. Faecal microbiota transplantation, FMT; rectal bacteriotherapy, RBT.

Through subgroup analyses excluding all patients who had previously received FMT or RBT ((aHR = 1.08; 95% CI 0.71–1.64), *p* = 0.72), for patients who received both FMT and RBT for their current episode of CDI ((aHR = 1.04; 95% CI 0.67–1.59), *p* = 0.87), patients who had received both FMT and RBT in the treatment for their current or previous episodes of CDI ((aHR = 1.07; 95% CI 0.69–1.65), *p* = 0.75) and patients who at any time (previously, currently and subsequently) received both FMT and RBT (aHR = 1.06; 95% CI 0.69–1.64), *p* = 0.78), no significant difference in survival was found between those treated with FMT or with RBT in the five years following treatment (Figures S1–S4). Comparing patients receiving FMT or RBT administered only through an enema, there was no difference in survival between the treated patients ((aHR = 0.99; 95% CI 0.65–1.53), *p* = 0.99) (Figure 3). There was also no difference in survival when comparing patients receiving FMT through an enema with those receiving FMT through capsules ((aHR = 0.99; 95% CI 0.38–2.55), *p*= 0.99) (Figure S5).

### 3.2. Onset of Disease and Disappearance of Disease/cessation of Treatment

Ten patients were referred for treatment of rCDI from the Region of Southern Denmark. It was not possible to access medical records in the Region of Southern Denmark; therefore, this group of patients was excluded from all analyses except for the analysis of survival.

#### 3.2.1. Cancer

**Figure 4:**
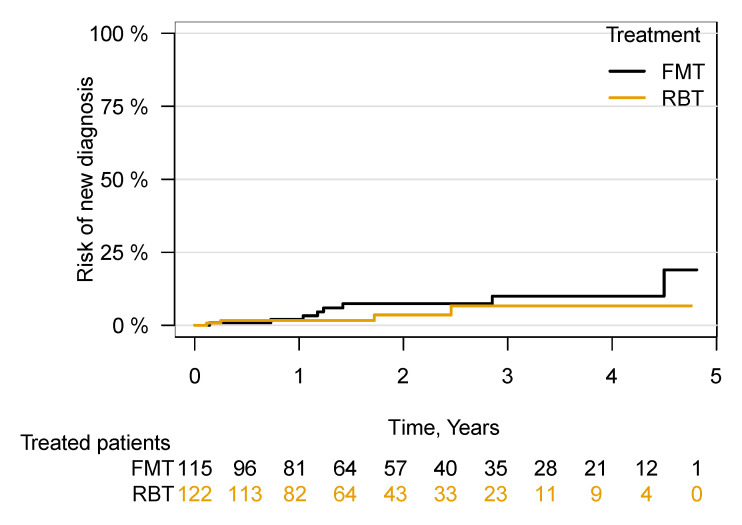
Risk of being diagnosed with cancer following FMT or RBT treatment for rCDI. Risk of being diagnosed with a new cancer in patients with no registered cancer at the time of first FMT/RBT. Faecal microbiota transplantation, FMT; rectal bacteriotherapy, RBT.

A total of 12 of the 237 patients (Table 2 and Table S1) not registered as having cancer at baseline were diagnosed with cancer in the follow-up period. No significant difference ((HR = 1.82; 95% CI 0.54–6.09), *p* = 0.33) between the groups was found (Figure 4). It was unaffected by the exclusion of patients who at any time (previously, currently or subsequently) received the other treatment to their primary treatment (FMT/RBT) ((HR = 1.61; 95% CI 0.47–5.43), *p* = 0.44). A single case of colorectal carcinoma was diagnosed 51 days following FMT treatment (Supplementary Table S1). All 31 patients with a cancer diagnosis at baseline still had cancer in the follow-up period (Table 3).

#### 3.2.2. Diabetes Mellitus

Five patients were diagnosed with DM2, with no significant difference between the groups following treatment ((HR = 3.44; 95% CI 0.38–31.02), *p* = 0.27) (Table 2). One patient ceased treatment for DM2 following RBT (Table 3). No new diagnoses of DM1 were recorded.

#### 3.2.3. Hypertension

A greater prevalence of patients who had received FMT were treated for hypertension at all time points (Figure S6). There was no significant difference between the groups in the risk of being diagnosed with hypertension following treatment ((HR = 2.32; 95% CI 0.42–12.7), *p* = 0.33) (Figure S7). Among the patients diagnosed with hypertension at baseline, there was no significant difference in the number of patients in terms of disappearance of disease or cessation of treatment (*p* = 0.58) (Figure S8).

#### 3.2.4. Inflammatory Bowel Disease

A single patient was diagnosed with possible Crohn’s disease 122 days following FMT treatment, identified in ulcers seen with capsule endoscopy, and began treatment with glucocorticoids. It has not subsequently been possible to confirm the diagnosis through endoscopy, biopsies or abdominal magnetic resonance imaging (MRI). No patients stopped treatment for their underlying IBD in the five years following FMT/RBT treatment.

#### 3.2.5. Multidrug-Resistant Organisms

A total of nine new cases of MDRO were diagnosed in the first six months following FMT or RBT treatment, most of them Vancomycin-resistant *enterococcus* (VRE), with no significant difference between the groups ((HR = 1.05; 95% CI 0.20–5.45), *p* = 0.96) (Table S2). Most of the new cases of registered MDRO were VRE. Only one patient had previously tested negative for VRE.

Among the twelve patients diagnosed with MDRO prior to treatment with FMT, two were registered with an absence of MDRO through new microbiological samples within the first six months of treatment (Table S3). In patients treated with RBT, there were no cases of MDRO disappearing in patients following treatment, while all six patients who were re-tested were still carriers of VRE (Table S4).

### 3.3. Hospital Admission

**Figure 5:**
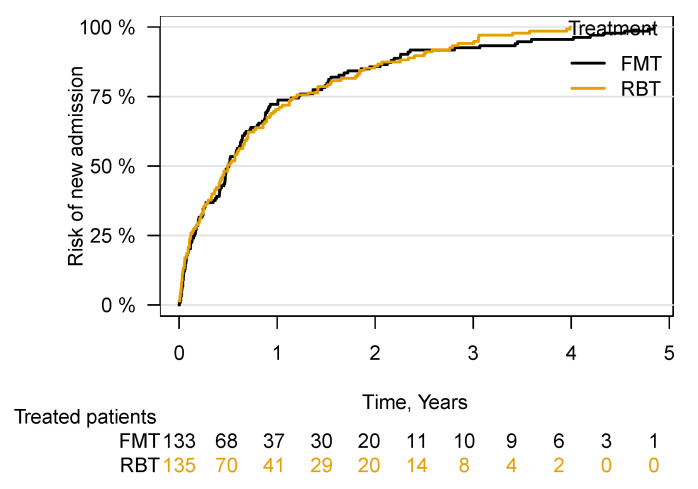
Time to first hospital admission following FMT or RBT treatment for rCDI. Faecal microbiota transplantation, FMT; rectal bacteriotherapy, RBT.

There was no increased risk of hospitalisation in patients following treatment with FMT compared with RBT ((aHR 0.92; 95% CI 0.72–1.18), *p* = 0.50) (Figure 5).

## 4. Discussion

In a long-term follow-up for up to five years of a large cohort of 280 patients treated for rCDI with FMT or RBT, no difference between treatments was found in terms of survival, risk of hospital admission, onset of IBD, hypertension or cancer, or transfer of MDRO. Furthermore, there was no difference in comorbidity between the two groups of patients prior to treatment.

The reported data are in accordance with previously published literature. The short-term safety of FMT in patients with rCDI is thoroughly established from the increasing number of patients treated worldwide and the results of RCTs [16,23,36]. Safety data from severely immunocompromised patients treated with FMT also point to an acceptable safety profile [37,38]. The most common short-term side effects of FMT treatment are gastrointestinal symptoms such as diarrhoea, bloating and abdominal cramping. The rare serious adverse events reported shortly after FMT treatment are primarily procedure related, but there have been two cases reported of a transfer of MDRO in neutropenic patients following treatment [14,16,17]. Given the retrospective set-up of this study, it was not possible to identify information on minor transient side effects.

Regarding long-term safety, FMT treatment has only been evaluated in a few studies, with no report of harmful effects [24,25]. Ooijevaar et al. (2021) recently published long-term safety data from the first RCT on FMT treatment for rCDI with patients followed for 11 years, and reported no long-term adverse effects or complications directly attributable to FMT [25]. Due to the lack of control groups in any of the previous studies on long-term safety following FMT treatment for rCDI, it is reassuring that the results reported here showed no increased risk of mortality, hospital admissions or cancer when compared with RBT treatment. Based on the superior results from FMT treatment compared with antibiotics, both in relation to clinical resolution of rCDI and survival, it appears unlikely that there will be future studies on the long-term safety of FMT based on RCTs with a control group receiving a placebo or antibiotics [26,39,40]. The findings reported here add to the available literature that indicates that there are few or no long-term harmful effects of FMT in patients treated for rCDI. Prospective studies with a longer follow-up are awaited to further determine long-term safety following FMT [41,42].

The primary strength of this retrospective cohort study is that safety data could be reported through the long-term follow-up of a large number of patients receiving FMT and the large group of patients who received RBT having a comparable control group. Furthermore, the Danish health care system, where survival is registered through cross-linkage to the Danish civil registration system, minimises the risk of bias when reporting data on survival. The registration of all hospital contacts into one system and the fact that the public hospital sector in Denmark accounts for the large majority of all treatments of most of the included diseases also minimises the chance of patients being omitted during follow-up when assessing the risks of new diagnoses following treatment.

This study has a number of major limitations. The non-randomised, retrospective set-up primarily means that some degree of selection bias cannot be ruled out, despite no reports of a difference in comorbidity between the treatment groups. However, the fact that no significant differences were found between the treatment groups at the group level may be due to the large heterogeneity of the treated patients with rCDI. Furthermore, the absence of data on clinical resolution following treatment limits the conclusions, since a fraction of the treated patients were re-treated with FMT or RBT due to treatment failure. However, when reporting data omitting patients who had received both FMT and RBT or had previously or subsequently received the opposite treatment, there was still no indication of harmful effects from any of the treatments in relation to survival.

When reporting the probability of the new onset or disappearance of the predefined diseases, there are a number of reasons why no firm conclusions can be drawn. These are primarily due to the fact that some of the diseases could have been diagnosed or treated in a primary health care setting and due to the retrospective design of the study, as treating physicians were not focusing in particular on the onset or disappearance of the selected diseases. Furthermore, the FMT dose used to treat rCDI was lower than the FMT doses that are often used in most studies to treat other diseases, where engraftment of a “healthy” donor gut microbiome is intended [9,43,44]. Hence, potential beneficial or harmful effects in relation to the defined diseases cannot be ruled out when long-term FMT treatment or larger doses are being used. Current evidence does not suggest an increased risk of serious adverse events in the treatment of diseases other than rCDI. However, there are still no studies that include a control group with long-term follow-up [11,45].

Another weakness of the study that should be noted is that most FMT treatments reported in this study were delivered through enemas. Hypothetically, the probability of inducing beneficial or harmful effects could be lower due to a smaller area of the gastro-intestinal tract being exposed to and colonised by donor microbes. The inclusion of FMT treatments supplied by various stool banks and delivered through different routes of administration also carries the risk of not identifying potential adverse effects caused by treatments from a particular stool bank or delivered through a specific route. 

FMT is known to induce major changes in the recipients’ gut microbiome with engraftment of donor bacteria that can be found in donors for up to five years following FMT [46]. The mechanism behind the successful treatment of rCDI following FMT is not fully understood [47,48]. Certain engrafting bacteria and viruses transferred through FMT have been correlated with a clinical cure of rCDI [49,50]. Engrafting bacteria affects many metabolic pathways in recipients. The beneficial role of engrafted bacteria has been correlated with bacterial conversion from primary to secondary bile acids, inhibiting *C. difficile* growth and toxin activity and leading to the production of short-chain fatty acids that also inhibit *C. difficile* growth [51,52]. Published studies reporting the results of RBT as a treatment for rCDI do not report changes in the gut microbiome following treatment; thus, changes induced by this treatment are not known [26,28,29,31]. Probiotic treatments are known to mainly affect the gut microbiome while treatment is being delivered, which might also be the case for RBT [53,54]. Thus, it is possible that RBT does not cause major long-term changes in the recipients’ gut microbiome to the same extent as FMT.

FMT has received considerable attention as a potential treatment for MDRO based on the beneficial effects reported by both case studies and RCTs [10,13,39,55,56]. Through data reported from just 16 patients with MDRO prior to treatment and no systematic follow-up, no difference in the clearance of MDRO in the FMT-treated patients compared with RBT-treated patients could be reported. FMT has also attracted interest as a treatment for VRE, which is becoming an increasing burden in several countries [55,57,58]. Since RBT also has microbiome-modulating potential in relation to MDRO, it cannot be regarded as a placebo. As VRE was found in microbiological samples in both patient groups following treatment, it is not considered to be correlated with the treatment for several reasons, the primary one being that the RBT treatment is thoroughly tested and all FMT stool banks supplying treatments to CUHH tested for this particular organism. The majority of patients were not tested for VRE prior to treatment. The risk of transfer of VRE increased in both the Capital Region of Denmark and in Zealand, from where the patients were referred in the study period, with increased testing carried out later in the study period [57,59]. Many of the treated patients had multiple comorbidities and frequent hospital contacts, hence the risk of transfer from sources other than FMT or RBT must be considered to be high [60,61].

The risk of inducing a flare up or new case of IBD following FMT in patients with rCDI is questionable since it is based on case studies and has not been reproduced in RCTs [16,19,36]. The reported results did not point to a correlation between FMT and induction of IBD. *C. difficile* is often diagnosed in patients with an underlying undiagnosed IBD or in a disease flare up in patients with underlying IBD, complicating establishment of the causality of symptoms [62]. FMT in the treatment of IBD has shown promising results without short-term safety risks when compared with a placebo; thus, further research should be encouraged within this field [21,63].

The working hypothesis of this study, that there may be increased long-term safety risks associated with the FMT treatment when compared with the RBT treatment, was not confirmed. These long-term safety data add to the current evidence that no long-term harmful effects are strongly correlated with FMT treatment. Data from larger cohorts and studies with longer follow-up are awaited to verify this. To further establish the safety profile of FMT, results from trials of diseases other than rCDI, where the treated patients are more homogeneous, are needed. It would be preferable to conduct placebo-controlled trials in which the patients are younger, have fewer co-morbidities and receive higher doses of FMT.

## 5. Conclusions

With this study, we found that the use of FMT to treat rCDI was safe in both the short and long term, with no sign of harmful effects, when compared with a control group receiving a fixed bacterial mixture as a treatment for rCDI. Mortality or the risk of being diagnosed with cancer, IBD, hypertension or diabetes following FMT did not increase, and no short-term risks of hospital admission or transfer of MDRO were identified. Further studies with long-term follow-up and studies of the treatment of other diseases are awaited to fully establish the safety profile of FMT treatment.

## Figures and Tables

**Table 1:** Baseline data of treated patients.

	FMT	RBT	*p*-Value
Number	145	135	
Age, mean (SD)	66.9 (18.8)	66.7 (19.7)	0.89
Sex:			
female	85	81	0.91
male	60	54	
Charlson Comorbidity Index, mean (SD)	4.3 (2.7)	4.4 (2.9)	0.69
Recurrences of CDI, mean (SD)	2.9 (1.5)	2.7 (1.3)	0.29
Location of treatment:			
outpatient	124	112	0.04
hospital admission	6	22	<0.01
at home	4	0	0.12
not recorded	11	1	
Previous FMT or RBT treatment (all delivered by enemas):			
FMT	2	3	0.67
RBT	2	0	0.49
both	2	1	1
Both FMT and RBT for current episode of CDI	5	21	<0.01
Cancer:			
all	18	13	0.41
colorectal	1	0	0.49
Diabetes mellitus:			
DM1	0	6	0.03
DM2	16	21	0.41
Hypertension	66	53	0.12
IBD:			
Crohn’s disease	1	5	0.21
Ulcerative colitis	12	10	0.79
IBD unknown type	1	1	1
MDRO:			
all	7	9	0.90
VRE	5	8	0.66
ESBL-producing *K. pneumoniae*	1	1	1
MDR *E. coli*	1	0	1

Faecal microbiota transplantation, FMT; rectal bacteriotherapy, RBT; standard deviation, SD; *C. difficile* infection, CDI; diabetes mellitus, DM; vancomycin-resistant *enterococcus*, VRE; extended spectrum beta-lactamase, ESBL; *Klebsiella pneumonia, K. pneumonia;* multidrug-resistant, MDR; *Escherichia coli, E. coli*.

**Table 2:** Onset of new diseases following treatment with FMT or RBT for recurrent *C. difficile* infection.

	FMT (New Diagnosis/Patients at Risk)	RBT (New Diagnosis/Patients at Risk)	*p*-Value
Cancer:			
first year	2/115	2/122	1
second and third year	5/80	2/82	0.27
fourth and fifth year	1/34	0/20	1
Multidrug-resistant organisms:			
first month	3/126	1/125	0.62
second to sixth month	4/120	1/124	0.35
Diabetes mellitus type 2:			
first year	2/117	0/108	0.5
second and third year	1/76	1/72	1
fourth and fifth year	1/31	0/19	1
Hypertension:			
first year	2/66	1/82	0.59
second and third year	1/44	1/51	1
fourth and fifth year	0/18	0/14	1
IBD:			
first year	1/113	0/119	0.49
second and third year	0/74	0/76	1
fourth and fifth year	0/29	0/22	1

Number of patients diagnosed with a new disease in patients not recorded with a diagnosis at time of FMT/RBT treatment. First year indicates all patients diagnosed with disease among patients who were followed for up to one year. Second and third year indicates all patients diagnosed with disease in the second or third year following FMT/RBT treatment among patients who were followed for more than one year. Fourth and fifth year indicates all patients diagnosed with disease in the fourth or fifth year following FMT/RBT treatment among patients who were followed for more than three years. The same principles apply for registration of multidrug-resistant organisms following treatment, but with different timeframes. First month indicates all patients diagnosed with MDRO in the first month following FMT/RBT and second to sixth month indicates all patients diagnosed with MDRO in the second to sixth month following treatment.

**Table 3:** Disappearance of disease/cessation of treatment following treatment with FMT or RBT for recurrent *C. difficile* infection.

	FMT (Disappearance of Disease/Patients with a Diagnosis)	RBT (Disappearance of Disease/Patients with a Diagnosis)	*p*-Value
Cancer:			
first year	0/18	0/13	1
second and third year	0/8	0/7	1
fourth and fifth year	0/3	0/3	1
Multidrug-resistant organisms:			
first month	0/7	0/9	1
second to sixth month	2/5	0/8	0.25
Diabetes mellitus type 2:			
first year	0/13	0/20	1
second and third year	0/10	1/11	1
fourth and fifth year	0/6	0/3	1
Hypertension:			
first year	2/61	4/49	0.49
second and third year	0/37	2/32	0.21
fourth and fifth year	0/17	0/14	1
IBD:			
first year	0/12	0/13	1
second and third year	0/8	0/10	1
fourth and fifth year	0/4	0/2	1

Number of patients where disease disappears for those registered with diagnosis at time of FMT/RBT treatment. First year indicates all patients where disease disappears out of patients who were followed for up to one year. Second and third year indicates all patients where disease disappears in the second or third year following FMT/RBT treatment out of patients who have been followed for more than one year. Fourth and fifth year indicates all patients where disease disappears in the fourth or fifth year following FMT/RBT treatment out of patients who have been followed for more than three years. The same principles apply for registration of disappearance of multidrug-resistant organisms following treatment, but with different timeframes. First month indicates all patients registered with disappearance of MDRO in first month following FMT/RBT and second to sixth month indicates all patients registered with disappearance of MDRO in the second to sixth month following treatment.

## Data Availability

The data supporting all the figures and tables in the published article are not publicly available due to restrictions. The authors do not have permission to share the data.

## Supplementary Materials

The following are available online at https://www.mdpi.com/article/10.3390/cells11030435/s1. Figure S1. Survival following treatment with FMT or RBT for recurrent *C. difficile* infection in patients previously not treated with FMT or RBT. Figure S2. Survival following FMT or RBT for recurrent *C. difficile* infection in patients treated with either only FMT or only RBT for current episode of *C. difficile* infection. Figure S3. Survival following FMT or RBT for recurrent *C. difficile* infection in patients treated with either only FMT or RBT for current episode and without previous treatment with opposite treatment. Figure S4. Survival following FMT or RBT for recurrent *C. difficile* infection in patients treated with either only FMT or RBT for current episode without previous or later treatment with opposite treatment. Figure S5. Survival following FMT or RBT for recurrent *C. difficile* infection in patients treated with either FMT capsules or FMT enema. Figure S6. Prevalence of hypertension in the treatment groups following treatment. Figure S7. Risk of getting diagnosed with hypertension following treatment. Figure S8. Chance of losing diagnosis with hypertension following treatment.Table S1. New cancer diagnoses following treatment with FMT or RBT. Table S2. New cases of MDRO following treatment with FMT or RBT. Table S3. Loss of MDRO following treatment with FMT or RBT. Table S4. Ongoing cases of MDRO following FMT or RBT. Supplementary methods.

## Graphics

Graphical abstract was created with Biorender.Com.
