# Supplementary material for: Long-Term Safety Following Faecal Microbiota Transplantation as a Treatment for Recurrent Clostridioides difficile Infection Compared with Patients Treated with a Fixed Bacterial Mixture: Results from a Retrospective Cohort Study
Source: Cells. 2022 Jan 27;11(3):435. doi: 10.3390/cells11030435 (PMC8834574; doi:10.3390/cells11030435)
Supplement: Supplementary file 1 [file cells-11-00435-s001.zip › cells-1494379-supplementary.pdf]

Supplementary figures and tables:

Long-term safety following faecal microbiota transplantation as a treatment for recurrent Clostridioides difficile infection compared with patients treated with a fixed bacterial mixture: Results from a retrospective cohort study

Table of contents

Table S1. New cancer diagnoses following treatment with FMT or RBT
Table S2. New cases of MDRO following treatment with FMT or RBT
Table S3. Loss of MDRO following treatment with FMT or RBT
Table S4. Ongoing cases of MDRO following FMT or RBT
Figure S1. Survival following treatment with FMT or RBT for recurrent *C. difficile* infection in patients previously not treated with FMT or RBT
Figure S2. Survival following FMT or RBT for recurrent *C. difficile* infection in patients treated with either only FMT or only RBT for current episode of *C. difficile* infection
Figure S3. Survival following FMT or RBT for recurrent *C. difficile* infection in patients treated with either only FMT or RBT for current episode and without previous treatment with opposite treatment
Figure S4. Survival following FMT or RBT for recurrent *C. difficile* infection in patients treated with either only FMT or RBT for current episode without previous or later treatment with opposite treatment
Figure S5. Survival following FMT or RBT for recurrent *C. difficile* infection in patients treated with either FMT capsules or FMT enema.
Figure S6. Prevalence of hypertension in the treatment groups following treatment
Figure S7. Risk of getting diagnosed with hypertension following treatment
Figure S8. Chance of losing diagnosis with hypertension following treatment
Supplementary methods:
Hypertension
Inflammatory bowel disease

Table S1. New cancer diagnoses following treatment with FMT or RBT

	**FMT** **New cases following treatment (days after treatment)**	**RBT** **New cases following treatment (days after treatment)**
**Pulmonary**	**380, 430**	
**Breast**	**1643**	
**Hematologic**		**91, 628**
**Malignant Melanoma**		**42**
**Colo-rectal**	**52**	
**Other Gastrointestinal**	**519**	**897**
**Neurologic**	**451**	
**Urinary**	**266**	
**Unknown primary tumor**	**1042**	

New diagnosis of cancer in the first five years following FMT or RBT treatment in patients not diagnosed with cancer at time of FMT/RBT treatment

Table S2. New cases of MDRO following treatment with FMT or RBT

	FMT	RBT
	New cases following treatment (days after treatment)	New cases following treatment (days after treatment)
VRE (rectal swab)	6 (19*, 23, 54*, 67*, 77* 128*)	1 (21*)
ESBL-positive *E. coli* (urine sample)	1 (27)	1 (86)

New diagnosis of MDRO in the first six months following FMT/RBT in patients either previously not tested or tested without MDRO in the three months prior to FMT/RBT treatment. *Patients not previously tested prior to FMT/RBT. Multi-drug resistant organism, MDRO; . Faecal microbiota transplantation, FMT; rectal bacteriotherapy, RBT; Vancomycin resistant *enterococcus*, VRE; Extended spectrum beta-lactamase, ESBL; *Escherichia coli, E. coli.*

Table S3. Loss of MDRO following treatment with FMT or RBT

	FMT	RBT
	Loss of MDRO following treatment (days after treatment)	Loss of MDRO following treatment (days after treatment)
VRE (rectal swab)	1 (63)	
ESBL-positive *K.- pneumonia* (urine sample)	1 (171)	

Loss of MDRO in the first six months following FMT/RBT in patients with a MDRO in a microbiological sample in the three months prior to FMT/RBT treatment that are re-tested in first six months following treatment. Multi-drug resistant organism, MDRO; . Faecal microbiota transplantation, FMT; rectal bacteriotherapy, RBT; Vancomycin resistant *enterococcus*, VRE; Extended spectrum beta-lactamase, ESBL; *Klebsiella pneumonia, K. pneumonia.*

Table S4. Ongoing cases of MDRO following FMT or RBT

	FMT	RBT
	Still MDRO in patients re-tested following FMT treatment (days after treatment)	Still MDRO in patients re-tested following RBT treatment (days after treatment)

VRE (rectal swab)	2 (6, 13)	6 (20, 37, 45, 69, 93, 131)
ESBL-positive *K.- pneumonia* (urine sample)		1 (58)

Still MDRO in a new microbiological sample from the first six months following treatment in patients with a MDRO in a microbiological sample in the three months prior to FMT/RBT treatment that are re-tested in the first six months following treatment. Multi-drug resistant organism, MDRO; Faecal microbiota transplantation, FMT; rectal bacteriotherapy, RBT; Vancomycin resistant *enterococcus,* VRE; Extended spectrum beta-lactamase, ESBL; *Klebsiella pneumonia, K. pneumonia*.

Figure S1. Survival following treatment with FMT or RBT for recurrent *C. difficile* infection in patients previously not treated with FMT or RBT

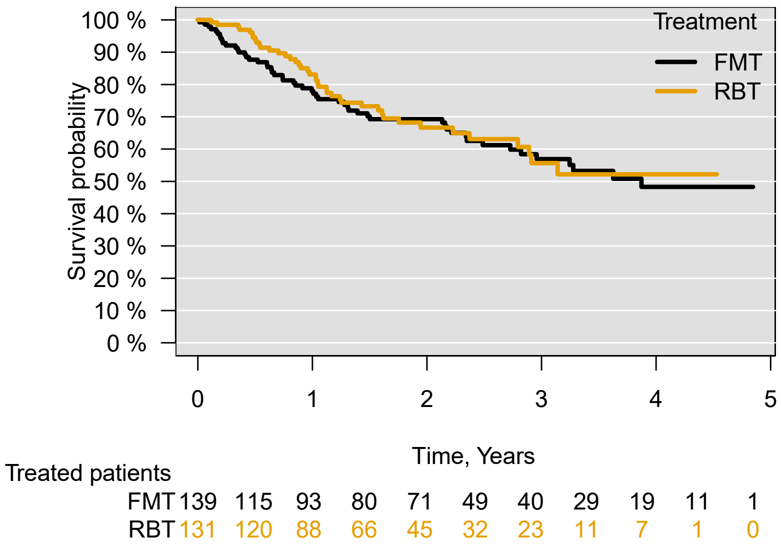

Figure S2. Survival following FMT or RBT for recurrent *C. difficile* infection in patients treated with either only FMT or only RBT for current episode of *C. difficile* infection

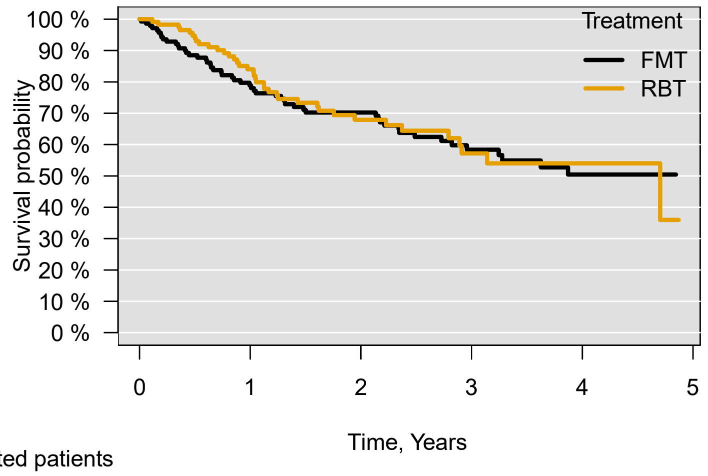

Treated patients

FMT	140	117	95	81	73	52	43	32	22	13	0
RBT	115	106	82	62	45	33	23	13	10	4	1

Figure S3. Survival following FMT or RBT for recurrent *C. difficile* infection in patients treated with either only FMT or RBT for current episode and without previous treatment with opposite treatment

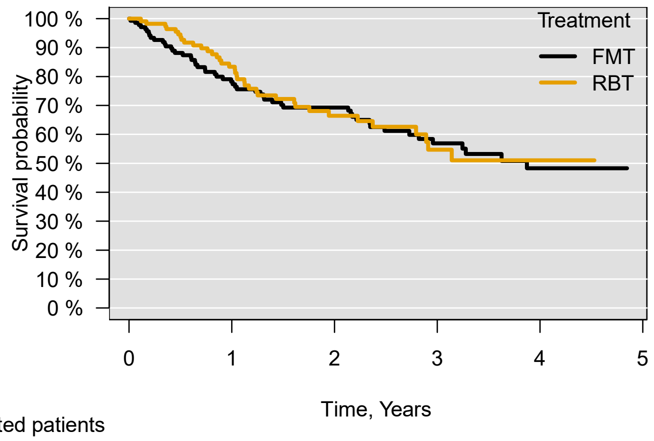

Treated patients

FMT	136	113	91	78	70	49	40	29	19	11	1
RBT	111	102	78	58	42	30	21	11	7	1	0

Figure S4. Survival following FMT or RBT for recurrent *C. difficile* infection in patients treated with either only FMT or RBT for current episode without previous or later treatment with opposite treatment

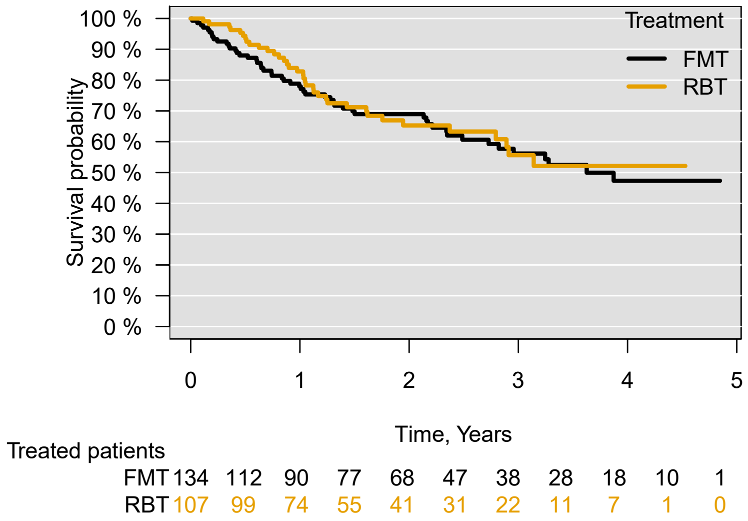

Figure S5. Survival following FMT or RBT for recurrent *C. difficile* infection in patients treated with either FMT capsules or FMT enema.

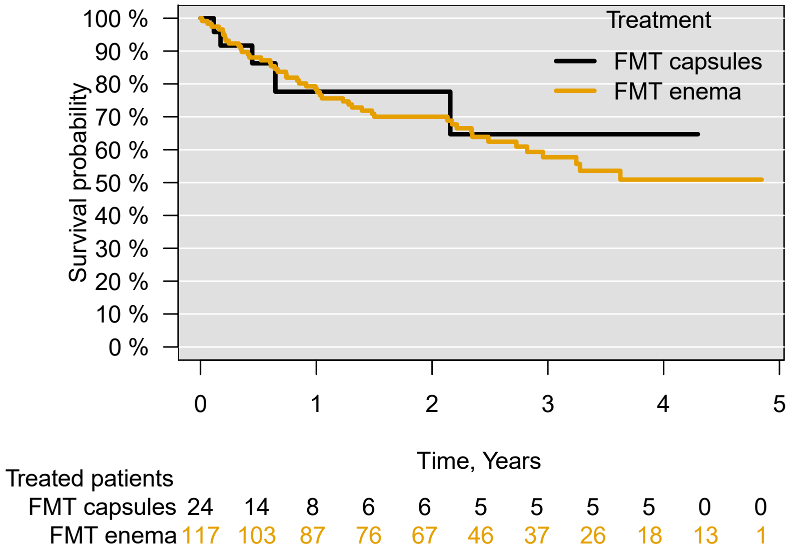

Treatment with FMT capsules were introduced in 2018 why no patients, were followed for more than four years.

Figure S6. Prevalence of hypertension in the treatment groups following treatment

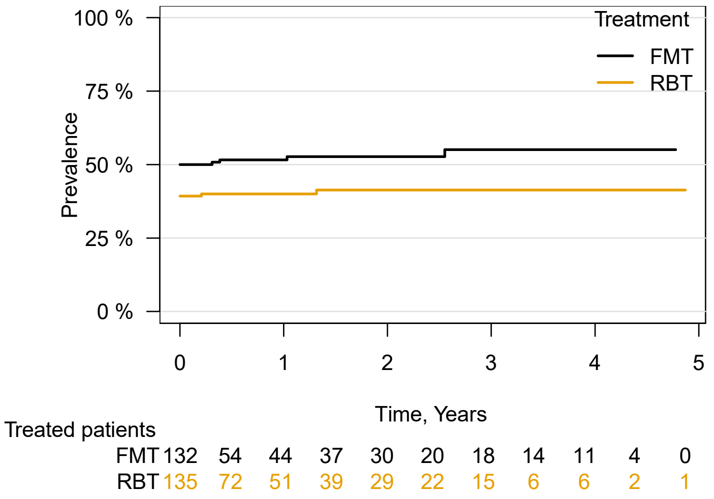

Figure S7. Risk of getting diagnosed with hypertension following treatment

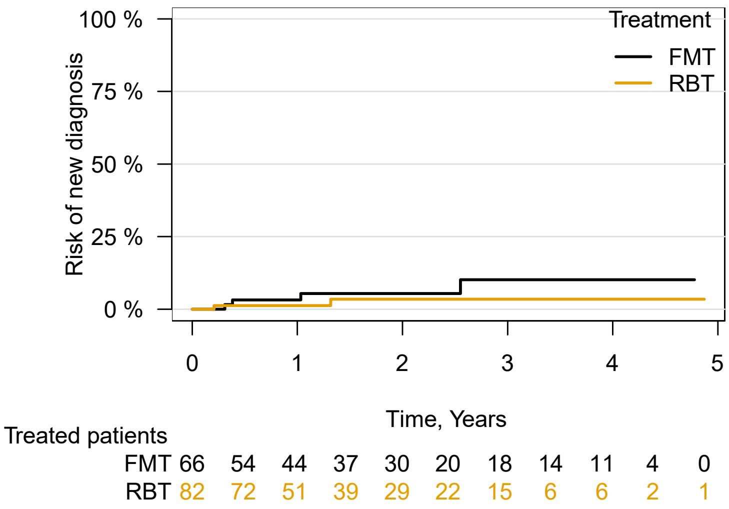

Figure S8. Chance of losing diagnosis with hypertension following treatment

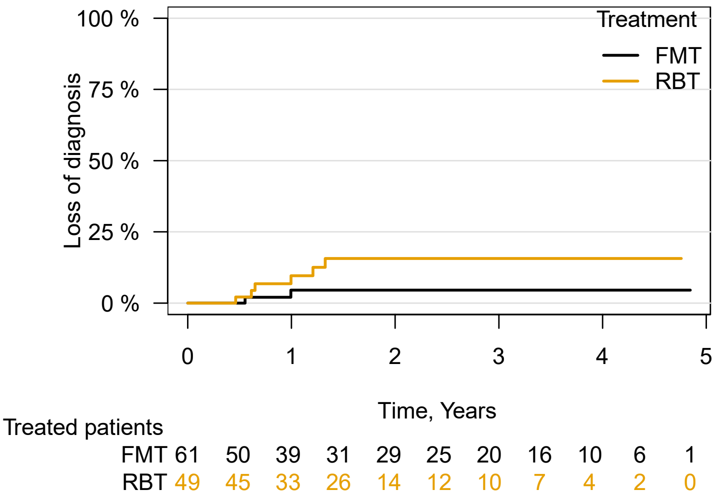

Supplementary methods

Hypertension

Presence of hypertension was based on active treatment of the disease based on registration of the diagnosis in the patient's medical record or treatment with the following antihypertensive medications; diuretics, adrenergic receptor agonists, ACE-inhibitors, angiotensin II receptor blockers, calcium channel blockers, vasodilators, renin-inhibitors, aldosterone receptor agonists, alpha blockers, beta-blockers, mixed alpha- or beta-blockers or alpha-2 adrenergic receptor agonists. In case of treatment with diuretics or medications also lowering cardiac rate a decision whether this was caused by other chronic diseases, e.g. kidney or liver disease or atrial fibrillation, was made based on the patient's medical record. Onset of disease following FMT or RBT treatment was based on the same criteria. Patients with hypertension are in Denmark primarily treated through their general practitioner and are only referred to hospital departments in cases of young age, accompanying symptoms or the need for several medications to regulate the blood pressure.

Inflammatory Bowel Disease

Presence of IBD was based on active treatment of the disease based on registration of the diagnoses Ulcerative Colitis, Crohns disease or unspecified IBD in the patient's medical record or treatment with the following medications; corticosteroids, aminosalicylates, azathioprines, mercatopurines, methotrexates or biological treatments. In case of treatment with the mentioned medications a decision whether this was caused by other chronic diseases was made based on the patient's medical record. Loss of disease/stopped treatment was based on a description of this in the patient's medical record and/or stopped treatment with all of the previously mentioned medications in patients without ongoing symptoms. Patients with IBD are primarily treated through hospital departments in Denmark.
